# Fluoride and gallein regulate polyphosphate accumulation in dental caries-associated Lacticaseibacillus

**DOI:** 10.1099/mic.0.001519

**Published:** 2024-11-28

**Authors:** Subhrangshu Mandal, Beverly E. Flood, Mark Lunzer, Dhiraj Kumar, Jake V. Bailey

**Affiliations:** 1 Department of Earth & Environmental Sciences, University of Minnesota – Twin Cities, Minneapolis, MN 55455, USA; 2 Department of Botany, Visva Bharati University, Bolpur, West Bengal, India; 3 Department of Diagnostic and Biological Sciences, University of Minnesota – Twin Cities, Minneapolis, MN 55455, USA

**Keywords:** caries, fluoride, gallein, PEP-PTS, polyphosphate

## Abstract

Inorganic polyphosphates (polyPs) are energy-storing biopolymers synthesized by all three domains of life. PolyP accumulation has been well studied with respect to its role in stress response, but its role in dental disease has received less attention. Dental decay can be promoted by changes in pH as well as the chemical activity of ions such as phosphate in oral fluids at the enamel interface. Previous work has shown that the drawdown of phosphate from biofilm fluids can alter the saturation state of oral fluids to thermodynamically favour mineral dissolution. The members of the Lactobacillaceae are known to accumulate polyP and play a role in early-stage and late-stage dental caries. In this study, we examined the effects of potential metabolic inhibitors on polyP accumulation in *Lacticaseibacillus rhamnosus*. We observed that two inhibitors of the enzyme responsible for polyP synthesis, gallein and fluoride, inhibited polyP accumulation in a balanced medium. However, fluoride and gallein treatments amended with either glucose or lactate were found to enhance polyP accumulation. These results illustrate the potential complexity of polyP metabolism in the oral environment.

## Importance

Polyphosphate (polyP) accumulation may play a synergistic role with sugar metabolism in the formation of dental decay. Fluoride has long been used as a preventative measure against caries, and our work suggests that fluoride may suppress polyP synthesis under some conditions, but under other conditions, such as sugar exposure, fluoride may instead increase polyP accumulation. Future studies may elucidate the complex interplay between biology and environmental factors that shape the influence of bacterial polyP accumulation on human health.

## Introduction

Dental caries is a global public health burden [1]. Mutans streptococci were once the primary focus of research on caries aetiology [2,4]. But more recently, the role of the broader microbial community in the oral environment is being investigated [5,9]. Regardless of the agent, the primary canonical mechanism of caries formation involves acid production by bacteria via the fermentation of sugars, which drives mineral dissolution [10]. The prevention primarily focuses on reducing sugar intake [11] and improving oral hygiene as well as employing fluoride treatments [12]. However, these approaches have been ineffective and/or untenable for many people, and newer approaches are being explored, including promoting positive synergistic microbial interactions while suppressing those that promote tooth decay [13]. The complexity of the oral microbiome, as well as its interaction with human health, is a significant challenge in unravelling these synergistic mechanisms.

Breiland *et al.* proposed a previously unrecognized mechanism that could also promote caries – the accumulation of intracellular polyphosphate (polyP) by oral taxa [14]. Similar to acidity, a reduction in the salivary concentration of the ions that make up the enamel mineral hydroxyapatite (e.g. calcium and phosphate) can lower the chemical saturation state of the saliva with respect to the mineral, thus encouraging its dissolution. In simple terms, one can think of dissolving table salt in water. If the concentration product of the sodium and chloride ions (ion activity product) is below the equilibrium constant for the mineral dissolution reaction, the salt crystals will tend to dissolve (the solution is said to be ‘undersaturated’), whereas if these ion concentrations are high (above the solubility product), the mineral phase will tend to precipitate (the solution is said to be ‘supersaturated’). Saliva commonly contains an overabundance of phosphate and calcium to prevent tooth demineralization. However, the reduction of one or both of these ions could shift the saturation state of oral fluids with respect to hydroxyapatite toward dissolution, a mechanism that could work in concert with acid production to attack dental enamel [14].

Long-chain polyP molecules are energy-storing biopolymers composed of inorganic phosphate (P_i_), which are typically accumulated intracellularly as granules. The capacity to synthesize and store polyP occurs broadly across all domains of life. Micro-organisms synthesize and accumulate polyPs in response to diverse stimuli, as reviewed by Rao *et al.* and Albi and Serrano [15,16], with new discoveries occurring regularly [17,20]. In the oral environment, microorganisms may accumulate polyP for a number of reasons, including its use as an alternative to ATP (e.g. as a means of phosphorylating glucokinase), or as a response to environmental stress such as acidity or starvation.

Breiland *et al.* [14] identified 61 oral genera with strong genetic potential to accumulate polyP and an additional 19 genera with variable genetic potential, including the members of the clade Lactobacillaceae (e.g. *Lacticaseibacillus rhamnosus* was formerly named *Lactobacillus rhamnosus*). Lactobacillaceae and other lactic acid bacterial (LAB) species, e.g. *Streptococcus mutans*, commonly lack respiratory pathways and are strict aerotolerant fermenters, although some LAB can carry out aerobic respiration when provided with haem [21]. Strains of *L. rhamnosus* have been isolated from ecologically diverse niches, including dairy products, fermented meat, vegetables, cereals, sewage, invertebrates and humans (oral, vaginal, intestinal and other clinical sources) [22,24]. Several strains of Lactobacillaceae have numerous clinically advantageous roles (acting as potential probiotics; e.g. *L. rhamnosus* GR-1) and are part of the indigenous human gut bacterial community [25,27]. Other strains could be associated with pathogenicity, particularly in the oral environment [28,30]. Indeed, a large number of lactobacilli, such as *L. rhamnosus*, *Lacticaseibacillus casei*, *Lacticaseibacillus paracasei*, *Lactobacillus acidophilus* and *Lentilactobacillus buchneri*, have been reported to be present in early- or late-stage dental caries [31,34].

Breiland *et al.* used growth studies and geochemical modelling to show that under certain conditions, the drawdown of orthophosphate (P_i_) resulting from polyP accumulation was sufficient to change the saturation state of oral fluids with respect to hydroxyapatite (supersaturated to undersaturated). In this study, we build on the work by Breiland *et al.* [14] by investigating the influence of potential polyP inhibitors and nutritional stimuli on polyP accumulation by *L. rhamnosus* ATCC 7469/DSM 20021. We conducted these studies using a brain heart infusion (BHI) growth medium because we found the medium best suited for studying a broad diversity of oral taxa. We examined the role of nutrient stimuli on polyP accumulation by measuring intracellular polyP sequestration and characterized the intracellular polyP via ^31^P NMR. Furthermore, we investigated the effects of two potential therapeutic inhibitors, gallein and fluoride, on polyP accumulation and examined the effects of nutrient additions on our inhibition assays. We also looked at the genetic potential of the Lactobacillaceae and report on potential biochemical interactions with gallein and fluoride. Finally, we used a geochemical modelling approach to estimate the potential effects of fluoride on the saturation state of hydroxyapatite and fluorapatite, the principal mineral constituents of dental enamel.

## Methods

### Growth assays

*Lacticaseibacillus rhamnosus* ATCC 7469 was obtained from the United States Department of Agriculture (USDA) Agricultural Research Service Culture Collection organization. For all the experiments, seed cultures of *L. rhamnosus* were prepared in BHI broth (BD BACTO BHI). A 1.0% (v/v) inoculum was then transferred from mid-log-phase seed cultures to the experimental treatments. A bacterial growth curve assay was performed under different treatment conditions to identify the actual physiological state where the maximum amount of P_i_ drawdown occurred. For this purpose, 1 ml of culture from different treatment culture sets was periodically taken to measure the OD_600_ value and pH. All replicates are represented as the mean of three culture sets. Cell counts for *L. rhamnosus* grown on BHI medium were performed in triplicate.

### Quantification of P_i_ uptake

The quantification of P_i_ from the extracellular medium was performed following the method originally described by Hansen and Koroleff [35], with some minor modifications. Briefly, 1 ml of culture was taken at select time points, centrifuged at 9500 ***g*** for 5 min, and concentrations of dissolved P_i_ in the supernatant were then measured using ascorbic acid-based assay (100 µl of ascorbic acid-mixed reagent to 1.0 ml of diluted cell-free extract).

### Isolation of intracellular polyP

Neutral phenol/chloroform and ethanol-based protocols were employed to isolate intracellular polyP, as originally described by Bru *et al.* [36], with some minor modifications. Briefly, bacterial cells were harvested from the mid-log to the stationary phase, where we recorded the maximum drawdown of P_i_ from our phosphate uptake experiments. The cell pellet was washed twice with 0.9% NaCl solution and resuspended in Sodium acetate 50mM and EDTA 10mM (AE) buffer (consisting of 50 mM sodium acetate and 10 mM EDTA) at 4 °C. The cell suspension was transferred into another solution containing 300 µl of saturated phenol and 40 µl of 10% SDS, vortexed, incubated at 65 °C for 15 min and then chilled for 2 min on ice. The supernatant was then transferred and mixed with 350 µl of chloroform. The whole mixture was then centrifuged for 2 min at 13 000 ***g***, and the aqueous phase was collected and treated with RNase and DNase treatment at 37 °C for 1 h. The reaction mixture was then added to 1 ml ice-cold ethanol and 40 µl of 3M sodium acetate and incubated for 6 h at −20 °C to precipitate the polyP. Finally, the polyP pellets were collected by centrifugation at 13 000 ***g*** at 4 °C for 15 min. The polyP pellets were washed twice with 70% ice-cold ethanol and dried at room temperature. Finally, the isolated polyP was dissolved in phosphate-free deionized Millipore water and stored at −20 °C for future use.

### Overexpression and purification of ScPPX enzyme for quantification of intracellular polyP

Plasmid pKM263-ScPPX, containing *Saccharomyces cerevisiae* exopolyphosphatase *ppx1* gene was procured from the plasmid repository Addgene (plasmid # 38327; http://n2t.net/addgene:38 327; Research Resource Identifier (RRID): Addgene_38 327) and used for the overexpression and purification of the ScPPX enzyme [37]. For this purpose, we have inoculated the *E. coli* strain BL21 (containing the pKM263-ScPPX plasmid) into 5 ml of Luria–Bertani (LB) broth medium containing 100 µg ml^−1^ ampicillin and incubated overnight in an incubator shaker at 250 r.p.m. at 37 °C. A 1% (v/v) inoculum (0.6 OD culture) was transferred into 100 ml of LB medium containing 100 µg ml^−1^ ampicillin and incubated for 3 h at 37 °C. When the bacterial culture reached the OD ≈0.6, 0.5 mM IPTG (MilliporeSigma) was added to induce ScPPX. The culture was incubated at 30 °C at 250 r.p.m. The bacterial cells were harvested by centrifugation at 4 °C at 20 000 ***g*** for 2 min. The pellet was resuspended in 5 ml of extraction/wash buffer (50 mM HEPES, pH 7.4, 300 mM NaCl). The whole cell suspension was then treated with lysozyme (10 mg ml^−1^, MilliporeSigma), RNaseA (10 mg ml^−1^, MilliporeSigma) and DNaseI (2000 U ml^−1^, New England Biolabs) and incubated at room temperature for 30 min of continuous shaking. The cell disruption was performed by sonication using a Fisher Dismembrator FB-120 at 20 kHz, on ice (5 min, 60% amplitude, 1/4′ probe and 10 s on/off). The lysate was then centrifuged for 20 min at 12 000 ***g*** at 4 °C. The supernatant was transferred to a 2.0 ml disposable column (Takara-Clontech) with 1 ml of TALON Superflow Resin (Takara-Clontech). At this point, columns were washed 3× with 5 ml of extraction/wash buffer (50 mM HEPES pH 7.4, 300 mM NaCl). An elution buffer (50 mM HEPES pH 7.4, 300 mM NaCl and 150 mM imidazole) was added to the column to elute ScPPX. ScPPX purity was further checked by using 4%/12% SDS-PAGE gel (Fig. S3, available in the online version of this article).

### Enzyme assay for polyP quantification

The extracted polyP was quantified following the method described previously by Pokhrel *et al.* [38], with some minor modifications. The ScPPX master mix was first prepared, which contained 100 mM Tris-HCl, 25 mM MgCl_2_ and 250 mM ammonium acetate (pH 7.5). Then, this mixture was added to 19 µl of diH_2_O and 1 µl of purified ScPPX (1 mg ml^−1^) and incubated for 20 min at 37 °C. The extracted polyP (100 µl) was mixed with 50 µl of reaction mixture. Each reaction mix sample set was further subjected to an ascorbic acid-based assay. Finally, polyP levels were expressed in terms of the concentration of individual polyP-derived free phosphate. Hence, all the resulting values were multiplied by 1.5 to calculate the mM concentration of polyP-derived orthophosphate, present in the entire extract. These polyP-derived free P_i_ were then finally expressed in micromolar concentrations.

### ^31^P 1D solution NMR of extracted intracellular polyP

*L. rhamnosus* was grown on semi-defined minimal medium as in Breiland *et al.* [14], incubated overnight at 37 °C and then harvested at an OD_600_ of 3.3. The extracted polyP and a polyP standard [sodium phosphate glass type 45 (Sigma-Aldrich)] were each eluted in 500 µl ddH_2_O, 10 mM EDTA and 10% deuterated water. The *L. rhamnosus* sample was measured for 16.35 h using an Avance III 400 NMR spectrometer operating at 161.97 MHz. The acquisition was performed at 298 K with a 3.38-s acquisition time and spectral width of 9689.923 Hz. The spectra were acquired using TopSpin 3.5 software (Bruker) with standard zgig pulse programming parameters.

### Visualization of polyP granules via fluorescence microscopy

Microscopic imaging-based detection of polyP was performed following the method used by Breiland *et al.* [14]. DAPI is a fluorescent stain that has a binding potential for both polyP and DNA, and the resultant complexes, the DNA-DAPI complex and the polyP-DAPI complex, exhibited distinct emission spectra (461 and 525 nm, respectively) when excited under 360 nm light. The polyP-DAPI complex was further characterized using custom band-pass filters (Chroma) (DNA-DAPI excitation/emission, 345/455 nm; polyP-DAPI excitation/emission, 415/550 nm). This emission wavelength shift subsequently resulted in the emission of a distinct yellow colour that was used to discriminate the polyP-DAPI complex from the DNA-DAPI complex [39]. Periodically, the cells were harvested from different treatment culture sets of *L. rhamnosus* and fixed for staining. All culture sets were subjected to ethanol fixation, transferred to a poly-l-lysine eight-well slide and air-dried. Twenty microlitres of DAPI (5 µg ml^−1^) was added to each sample and then incubated in the dark for 30 min at room temperature in a hybridization chamber. Imaging was performed using an Olympus BX61 fluorescence microscope equipped with an XM10 charge-coupled device camera and CellSens Dimensions imaging software (v.1.13). Photoshop CS5 (v.12.0.4) was used to adjust the brightness and contrast uniformly across the entirety of the images.

### Saturation state solubility modelling methods

Saturation state modelling of hypothetical saliva with respect to hydroxyapatite and fluorapatite was performed using PHREEQC 3.0 [40]. The concentrations of ions [Ca^2+^] and [PO_4_^−^] and the pH conditions used for the modelling analysis were derived from Crea *et al.* [41]. The fluoride ion [F^−^] concentration was set at 2.6 µM based on measurements of 0.2 to 0.8 ppm [F^−^] in dental plaque fluid in the oral environment [42].

### Genetic analyses

The sequence of polyP metabolism-related proteins (e.g. polyP kinase, exopolyphosphatase and ABC family polysaccharide polyP export system-permease protein) was retrieved from the whole-genome sequence of *Lacticaseibacillus rhamnosus* ATCC 7469 (DSM 20021). Gene sequences related to PPK and PPX proteins that were used in this study were all further retrieved from the National Center for Biotechnology Information (NCBI) protein database (https://www.ncbi.nlm.nih.gov/protein/https://www.ncbi.nlm.nih.gov/prote). Each sequence that was retrieved in this way from the NCBI was then primarily checked using the NCBI conserved domain search [43] to eliminate the sequences that did not constitute relevant functional domains. The assessment of the similarity percentage and identity of the sequence with homologues of other PPK protein sequences has been done using Water Pairwise Sequence Alignment (EMBOSS) [44] (http://www.ebi.ac.uk/Tools /psa/embos s_water). Protein–chemical interaction networking was performed using STITCH [45]. The assessment of the genetic potential of oral Lactobacillaceae associated with caries to accumulate polyP was performed using annotated genomes in the Integrated Microbial Genomes (IMG) database [46].

## Results

### Effect of nutritional stimuli on P_i_ uptake and sequestration of polyP by *L. rhamnosus*

**Fig. 1.**
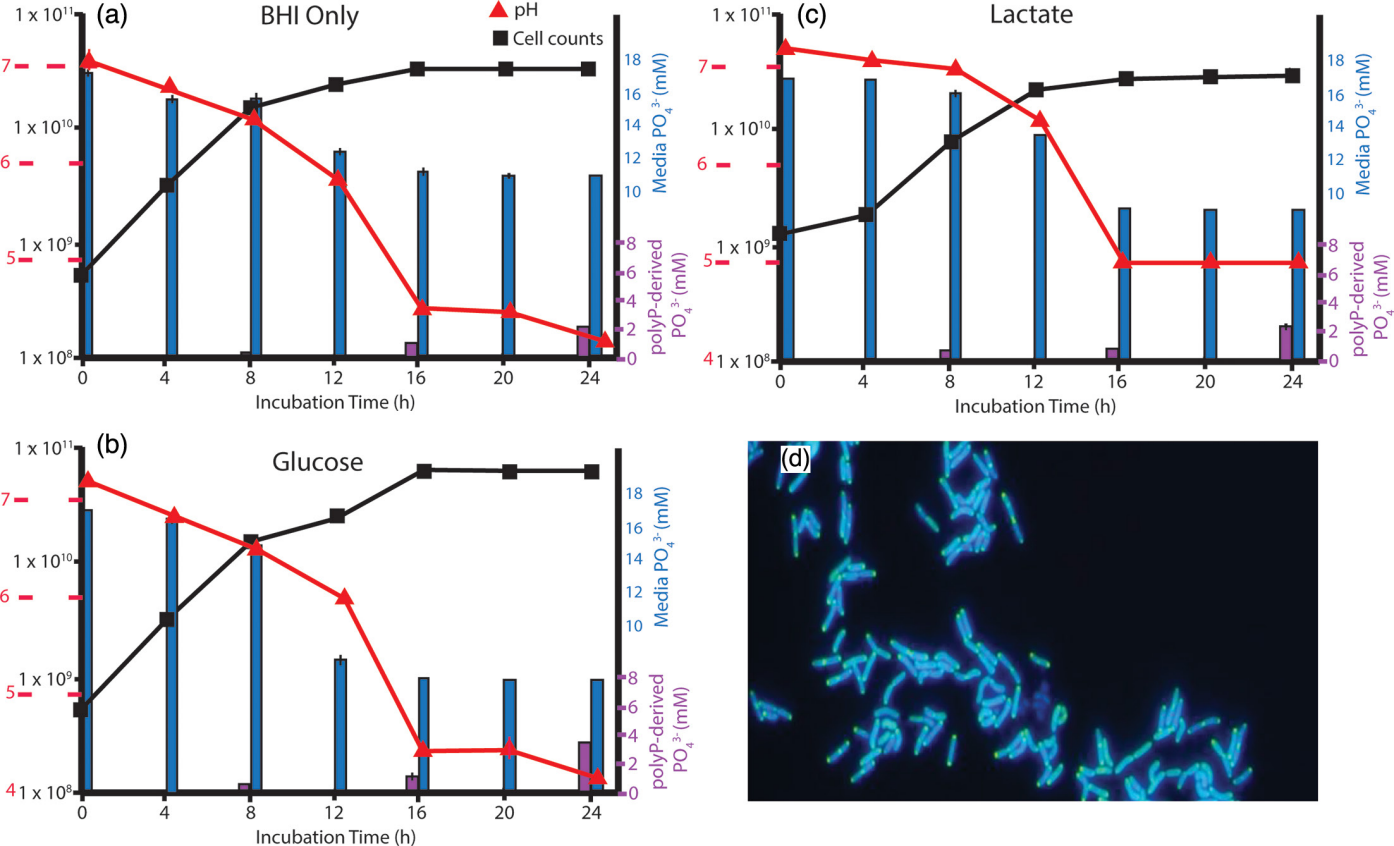
The effects of nutrient stimuli on Pi uptake and polyP sequestration by *L. rhamnosus*. The effects of nutrient amendments on the drawdown of P_i_ from the media (blue), the accumulation of intracellular polyP (purple) and changes in pH (red) were measured by growing *L. rhamnosus* on BHI and amended BHI media. (**a**) BHI only. (**b**) Addition of 50 mM glucose. (**c**) Addition of 10 mM lactate. Values plotted represent the mean and sd of measurements from three culture sets. The presence of polyP (yellow/green granules) in DAPI-stained *L. rhamnosus* cells (blue) was visually confirmed using fluorescence microscopy (**d**).

In order to examine the influence of inhibitors on polyP accumulation, we first quantified polyP accumulation by *L. rhamnosus* in the absence of potential inhibitors by measuring P_i_ removed from the media as well as P_i_ evolved from the enzymatic treatment of cells with exopolyphosphatase, an established method for quantifying intracellular polyP [47]. When grown on unamended BHI, 6 mM of P_i_ was taken up by *L. rhamnosus* from the media, predominantly during the mid to late-exponential phase of growth, when the pH had declined below 6 (Fig. 1a). The maximum polyP recovered from cell extracts (2 mM) using exopolyphosphatase treatment was quantified in the stationary phase, representing ~1/3 of the P_i_ consumed (6 mM). Under 50 mM glucose supplementation, we observed an increase in P_i_ (≈10 mM) uptake from the medium, concurrent with a period of diauxic growth and a decline in pH below 6 (Fig. 1b). This treatment exhibited the lowest stationary phase pH (4.4). Approximately 1.4 mM more polyP was measured in cell extracts from the glucose-amended experiment compared to unamended BHI as a control. The 10 mM lactate treatment also showed an increase in maximal P_i_ uptake (≈8 mM) when compared to the BHI-only control, but only slightly more intracellular polyP was detected (2.4 mM) (Fig. 1c). Similar to the glucose treatment, the cells in the lactate treatment exhibited diauxic growth, but lactate did not result in an increase in biomass, nor did it stimulate acid production. Indeed, the final pH in this treatment was higher than the pH of the BHI controls, whereas the overall biomass yield was lower than the BHI controls. In general, the late logarithmic phase and early stationary phase were found to be the stages in the growth cycle where the maximal amount of P_i_ was taken up from the medium. Microscopy of DAPI-stained cells with filter sets that capture the longer wavelength emission of the DAPI-polyP complex confirmed the maximum polyP accumulation during this stage (Fig. 1d).

### ^31^P NMR on polyP extracts

Along with DAPI staining and PPX enzyme-based P quantification, we used ^31^P NMR as a tertiary means of verifying the presence of polyP. The ^31^P NMR spectra for polyP extracted from *L. rhamnosus* exhibited characteristic peaks of polyP and were consistent with those of our polyP standard (Fig. S1). A peak at ~−5.8 represents the terminal phosphate group of the polyP chain (PP1). The peak at −21.72 (PP4) represents the core phosphate groups. The second and third terminal phosphate (PP2 and PP3) groups lie closer to −21 but were not well resolved. PolyP in bacteria can occur in a range of different chain lengths. Based on the formula used by Pilatus *et al.* [48] to calculate polyP length, the average chain length of linear polyP in our *L. rhamnosus* was ~646 phosphate groups indicating predominantly longer chain lengths in these cells. No other polymer chain forms of polyP were observed, which could be related to our extraction procedure [49]. In addition to peaks corresponding to polyP, four additional peaks were observed that have been seen in other ^31^P NMR spectra from LAB under sugar starvation [50,52]. The additional peaks were identified as phosphoenolpyruvate (PEP), 2-phosphoglycerate (2-PGA), 3-phosphoglycerate (3-PGA) and P_i_.

### The effect of the inhibitor gallein on the drawdown of media P_i_ and polyP accumulation

**Fig. 2.**
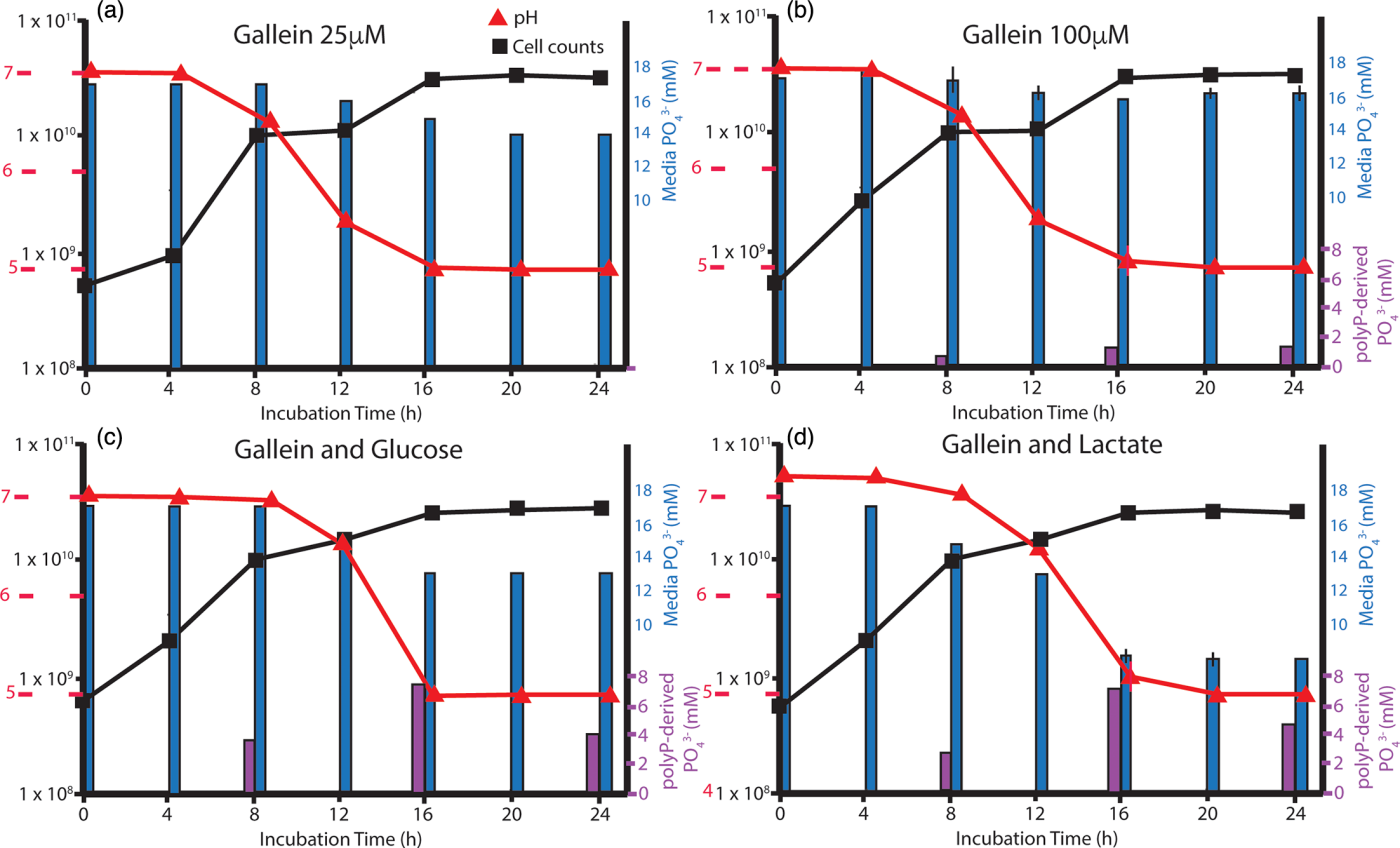
The effects of gallein on Pi uptake and polyP sequestration by *L. rhamnosus*.The cultures of *L. rhamnosus* (ATCC 7649) grown in the presence of the polyP inhibitor gallein exhibited a reduction in polyP accumulation in unamended media (**a, b**), but glucose and lactate additions in the presence of gallein did not show a reduction in polyP accumulation (**c, d**). Values plotted represent the mean and sd of measurements from three culture sets. (**a**) Addition of 25 µM gallein. (**b**) Addition of 100 µM gallein. (**c**) Addition of gallein and 50 mM glucose. (**d**) Addition of gallein and 10 mM lactate.

Gallein (pyrogallol phthalein) is a small polyphenolic molecule known for its *in vitro* inhibitory activity at low micromolar concentrations against bacterial polyP kinases that assemble long-chain polyP [53,54]. A few studies have explored the use of gallein using model systems, such as *Escherichia coli* or *Pseudomonas aeruginosa*, but not with oral taxa or gram-positive bacteria [53,55, 56] with the exception of the oral isolate *Rothia dentocariosa* [57]. Gallein was shown not to disrupt the community of commensal gut microbes [56], making it a promising therapeutic agent. Here, we investigated how gallein, both alone and in conjunction with glucose and lactate additions, influences the P_i_ drawdown capacity of *L. rhamnosus*. First, we examined the effects of two different concentrations of gallein (25 µM and 100 µM) in BHI media (Fig. 2a, b). The 25 µM and 100 µM treatments resulted in a reduction of P_i_ uptake by the bacterial cells relative to that observed without the addition of gallein (Fig. 2a, b) by ~50 and 85 %, respectively. While the growth curve cell densities were similar between gallein-treated and gallein-free experiments, a period of slower growth was observed in the mid-log phase at 8–12 h. Also, there was a very modest reduction in the total cellular yield as well as in acidity produced. In addition, the 100 µM gallein treatment resulted in an ~60% decrease in extracted polyP in the stationary phase (1.2 mM) compared to the unamended BHI.

We also supplemented 100 µM gallein with glucose or lactate to investigate whether nutrient stimuli that promote P_i_ drawdown would also result in lower uptake of P_i_ during combination exposures (gallein-glucose and gallein-lactate) (Fig. 2c, d). We observed that the inhibition of P_i_ uptake by gallein alone was removed by the addition of lactate and, to a lesser extent, by glucose. When comparing BHI additives such as gallein and lactate with lactate alone, the growth yield was slightly reduced, but the overall drawdown of P_i_ increased in the gallein treatment by 20%. Moreover, the total yield of polyP increased approximately threefold. The differences between gallein-plus-glucose amended BHI and glucose-only BHI (Fig. 1b) were even more substantial. The addition of gallein decreased both the total cellular yield and consumption of P_i_ by ~50%. However, the total polyP yield increased by more than 50%. When nutrient stimuli were added along with gallein, greater abundances of polyP were detected earlier in the growth phase than in the stationary phase.

### The effect of the inhibitor fluoride on polyP accumulation

**Fig. 3.**
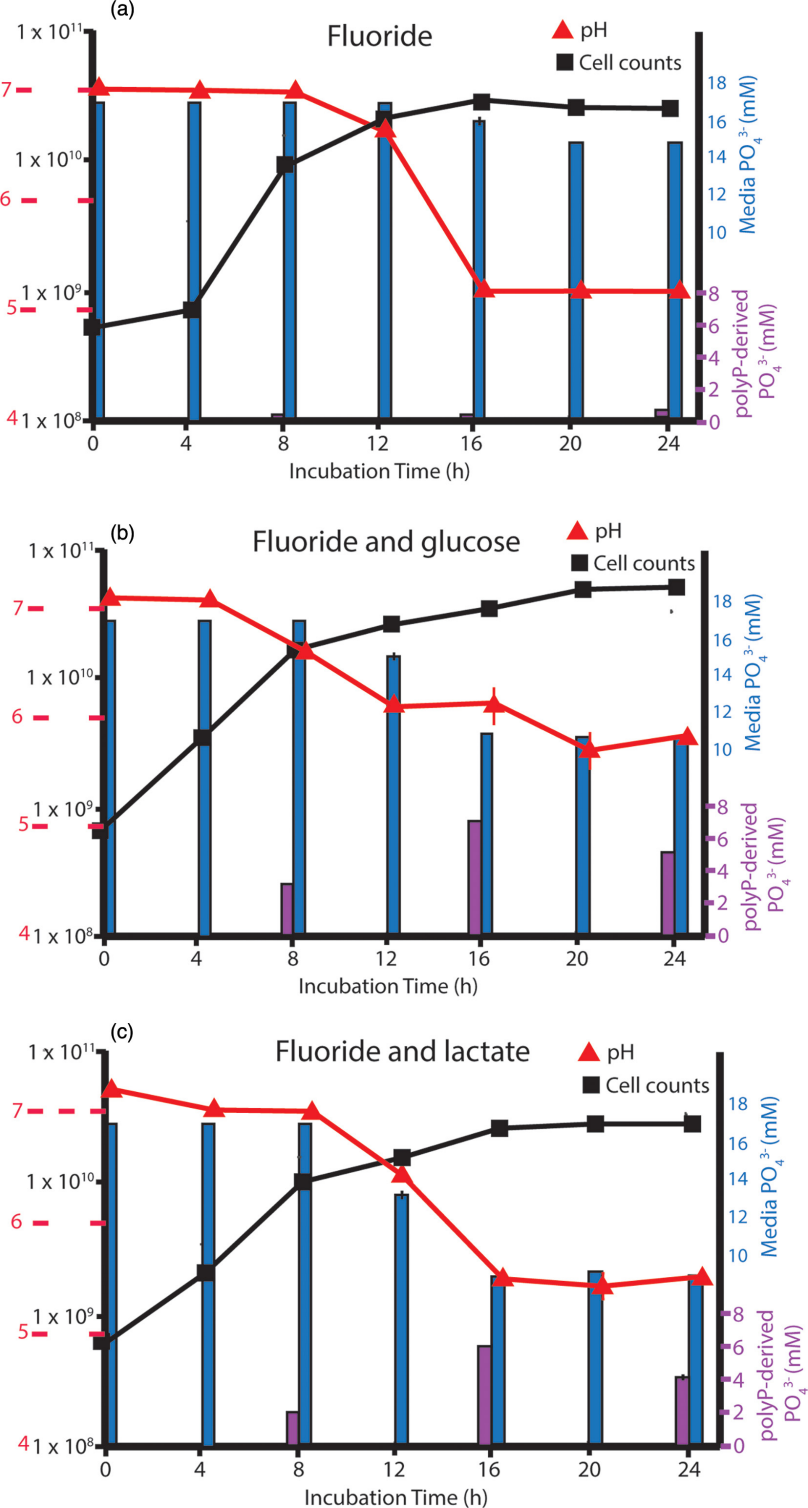
The effects of fluoride on Pi uptake and polyP sequestration by *L. rhamnosus*. As with gallein, fluoride reduced polyP accumulation by *L. rhamnosus* (ATCC 7649) grown in unamended BHI media. However, this inhibitory effect was reversed by the addition of glucose and lactate. Values plotted represent the mean and sd of measurements from three culture sets. (**a**) Addition of 1 mM fluoride only. (**b**) Addition of 1 mM fluoride and 50 mM glucose. (**c**) Addition of 1 mM fluoride and 10 mM lactate.

Fluoride exhibits cytotoxicity that is directly related to permeating cells as a weak acid [58,59]. Fluoride is also a known inhibitor of many enzymes that catalyse phosphoryl transfer reactions, predominantly by interacting with metal co-factors [59,61]. Likewise, *in vitro* studies on *E. coli* and gram-positive *Arthrobacter atrocyaneus* demonstrated that the primary enzyme for assembling long-chain polyPs, polyP kinase I, is inhibited by fluoride [62,63]. Fluoride supplementation has long been used to improve oral health, ostensibly because of its role in decreasing enamel mineral solubility. Therefore, we explored its potential as an inhibitor of polyP synthesis *in vitro*. When grown on BHI medium in the presence of 1 mM fluoride, two-thirds less P_i_ was taken up (~2 mM P_i_) during the exponential phase, concurrent with a decline in media pH to 5.2 (Fig. 3a). The growth curve data indicated that fluoride appears to modestly inhibit the growth of *L. rhamnosus* in addition to inhibiting acid production. Fluoride addition to BHI resulted in the greatest reduction in P_i_ uptake from the media and the greatest reduction in polyP accumulation (0.4 mM in stationary phase) observed in all treatments in this study.

However, as was observed in the gallein treatments, the addition of glucose and lactate in the presence of fluoride enhanced polyP accumulation. In the fluoride-plus-glucose treatment, there was a slight inhibition of growth in comparison to the glucose-alone treatment and 40% less P_i_ uptake (Fig. 3b). The addition of fluoride also significantly reduced the amount of acid produced. However, polyP accumulation increased by ~50% with the addition of glucose, and the accumulation occurred earlier in the growth cycle. The addition of lactate to the fluoride experiments revealed that fluoride did not inhibit bacterial growth or P_i_ drawdown in the presence of lactate (Fig. 3c). With the lactate addition, we observed moderately less acid production, while the polyP quantified was more than twice that observed in the lactate-only treatment and was again detected earlier in the growth phase.

### Genetic insights into the inhibitor interactions with *L. rhamnosus* ATCC 7469/DSM 20021

We analysed the genome of *L. rhamnosus* ATCC 7469 to explore the potential interactions between biochemical pathways and gallein and fluoride. Previous studies using gene disruption of *ppk1* in *L. casei* demonstrated that PPK1 is primarily responsible for polyP accumulation in Lactobacillaceae [45]. As previously reported [45], this strain has an operon containing a polyP kinase (type 1) *(ppk1*, KRK31516.1) flanked on either side by an exopolyphosphatase (*ppx1*, *ppx2*, KRK31515.1 and KRK31517.1, respectively). In addition to *ppk1*, *L. rhamnosus* possesses a class III *ppk2* gene (KRK31991.1; annotated as a hypothetical gene by GenBank). Class III PPK2 enzymes are a relatively new discovery [64] and are thought to phosphorylate mono- and diphosphate nucleosides, as reviewed by Neville *et al.* [54]; however, polyP synthesis can occur through this enzyme via adenosine monophosphate (AMP). The *ppk2* gene is not in an operon; thus, the gene arrangement is not insightful. PPK2 has been poorly studied in the Lactobacillaceae, but a PPK2 in *Limosilactobacillus reuteri* was found to play a protective role against reactive oxygen species [61].

### Genetic potential for polyP accumulation in oral Lactobacillaceae

Since polyP kinases are responsible for polyP accumulation, we explored how often they occur in Lactobacillaceae genomes of clades associated with dental caries [34]. A query of the IMG database [46] revealed that with the exception of the probiotic species *L. salivarius* as well as *L. gasseri*, Lactobacillaceae clades associated with caries have the genetic potential to accumulate polyP (Table 1). Considering that some of these annotated genomes are incomplete, nearly all Lacticaseibacilli (*L. rhamnosus*, *paracasei* and *casei*) possess *ppk1* and, to a lesser extent, *ppk2* genes.

**Table 1.** Presence of polyP kinases in clades of Lactobacillaceae associated with dental caries

Clade	TIGR3705*ppk1*	TIGR3709*ppk2*	Clade	TIGR3705*ppk1*	TIGR3709*ppk2*
*Lactobacillus (para)gasseri*	1/25	1/25	*Ligilactobacillus salivarius*	0/59	0/59
*Lacticaseibacillus* spp.	351/356	315/356	*Limosilactobacillus fermentum*	51/52	49/52
*Lactoplantibacillus plantarum*	329/331	326/331	*Limosilactobacillus mucosae*	9/9	9/9
*Lentilactobacillus (para)bucheri*	17/17	17/16	*Limosilactobacillus oris*	4/4	4/4
*Levilactobacillus (para)brevis*	50/50	50/50	*Limosilactobacillus vaginalis*	2/2	2/2

Numbers indicate genomes that include *ppk* genes/total number of queried genomes in the IMG database.

### Modelling results

**Fig. 4.**
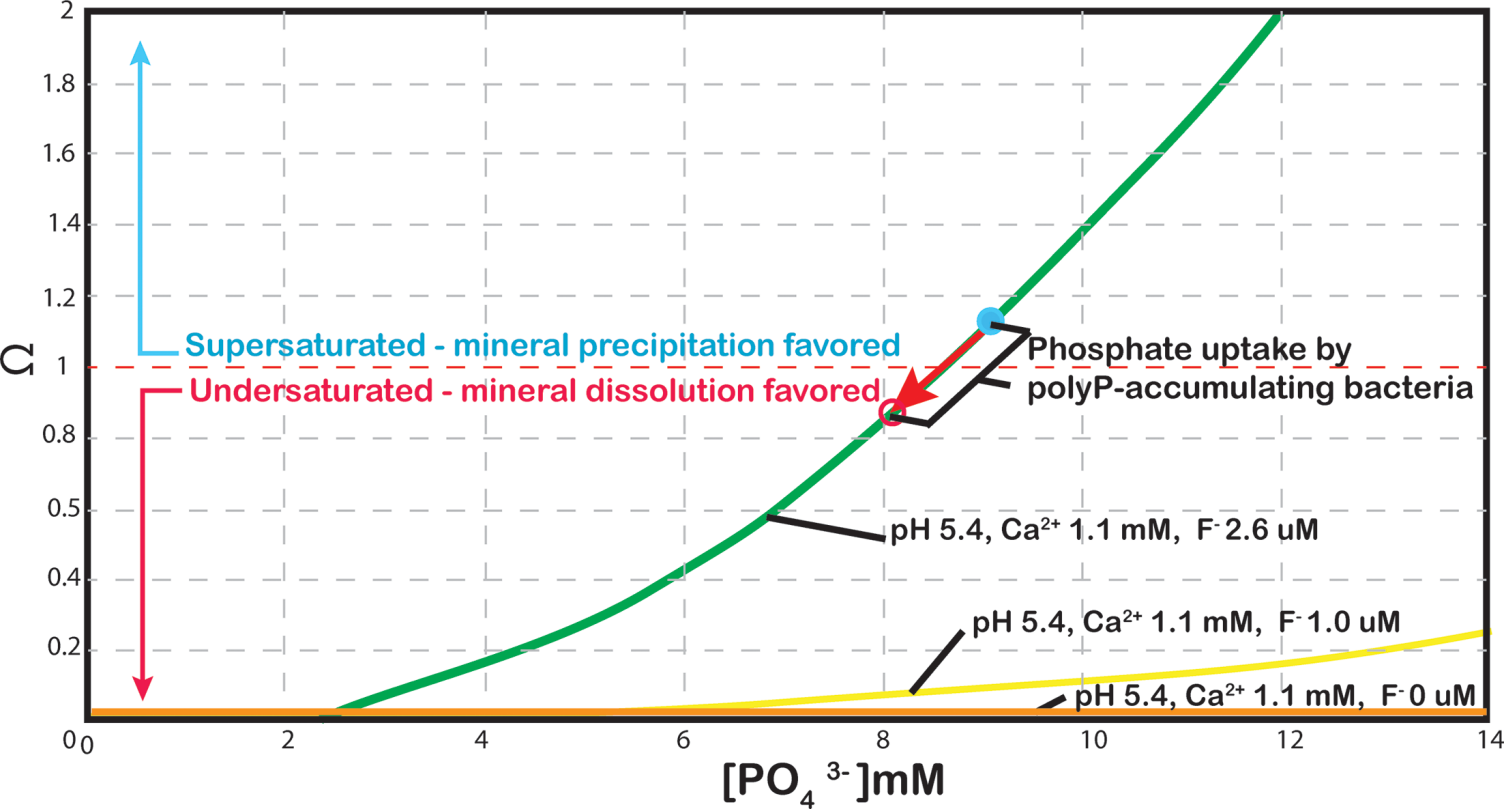
Saturation state modelling of hypothetical oral fluids. Saturation state modelling with respect to hydroxyapatite shows that polyP-accumulating bacteria can shift the saturation state (y-axis, omega) towards dissolution, red arrow, when sequestrating P_i_ (x-axis) at concentrations observed in this study.

We modelled the mineral saturation state (Ω) of dental plaque fluid with respect to hydroxyapatite over a pH range from 5.7 to 5.48 in increments of 0.2 mM for 0.2–14 mM (mmol l^−1^) for PO_4_^2−^ (Fig. 4). Ion concentrations were based on measurements of dental plaque fluid, as reported previously [41]. Under conditions when calcium ion concentrations are relatively low and fluoride is present, hydroxyapatite remained above saturation at phosphate concentrations of approximately 8.5 mM (Fig. 4, green line) and mineral precipitation would be favoured. Under conditions where fluoride is not present, the fluid is undersaturated at these calcium and pH conditions (Fig. 4, orange line), which would favour mineral dissolution. Phosphate uptake by polyP-accumulating bacteria can shift the saturation state of dental plaque fluids towards undersaturation in cases where conditions are near saturation (red arrow).

## Discussion

### The role of nutrient metabolism in polyP accumulation by *L. rhamnosus*

In order to investigate the role of the potential inhibitors fluoride and gallein, on polyP accumulation, we first examined the metabolism of *L. rhamnosus* on BHI media without the presence of inhibitors to establish a baseline for comparison. We also investigated the role of nutritional stimuli, glucose and lactate, on polyP accumulation because dietary factors such as sugar are known to be associated with the development and advancement of caries. In general, LAB can produce lactic acid as the major fermentation product of homofermentation or heterofermentation pathways [65]. The members of the former *Lactobacillus casei* group, which includes strains of *L. rhamnosus*, tend to perform facultative heterofermentation, whereby hexoses are fermented via a homofermentative pathway that utilizes the glycolytic pathway (Embden–Meyerhof–Parnas pathway), while pentoses are fermented via a heterofermentative pathway [66]. Homofermentation transforms 1 mol of glucose into pyruvate along with the production of two moles of lactic acid. Generally, lactate is not a preferred substrate when compared to sugars and is usually effluxed from the cell to maintain a proton motive force, but it can later be used by lactate dehydrogenases. In our experiments, the addition of glucose and lactate both increased polyP accumulation.

In addition to confirming the presence of polyP, our ^31^P NMR analyses detected several metabolites including PEP, 2-PGA and 3-PGA. During homofermentation of hexoses, extracellular pools of P_i_ mediate intracellular pools of Embden-Meyerhof-Parnas (EMP) intermediates. Upon sugar starvation, the transport of hexoses switches from active transport to a PEP-dependent phosphotransferase system (PTS), which phosphorylates the hexose upon entry into the cell, as reviewed by Thompson [67]. Nutrient-deprived LAB are poised to utilize the PEP-PTS system in conjunction with glycolysis by upregulating the production of PEP, 2-PGA and 3-PGA, as well as accumulating P_i_. *L. rhamnosus* possesses a complete glycolytic pathway and a large suite of PEP-dependent PTS transporters (KRK31612.1 and KRK30621.1).

### Potential relevance to oral fluids

The change in the saturation state of oral fluids from conditions that favour mineralization to those that promote demineralization depends on pH, as well as the concentration of calcium, phosphorus and fluoride in oral fluids (reviewed by Cury and Tenuta [68]). The primary canonical causative agent of caries is acid production via the fermentation of sugars such as sucrose. Sucrose can be converted into glucans and fructans, which form the building blocks of insoluble extracellular polymeric substances (EPS) and inhibit the flow of charged ions and molecules in and out of an oral biofilm [69]. However, numerous studies have observed a reduction of P_i_, calcium and fluoride in the biofilm matrix following a ‘sugar challenge’ [70,80]. While it was recognized that the loss of these ions in the biofilm fluids likely plays an uncharacterized role in demineralization, the previously proposed mechanisms for ion loss could only explain the loss of calcium and fluoride (e.g. decreased extracellular binding by a decrease in cells or calcium-binding proteins) [81]. These earlier studies measured P_i_, calcium and fluoride following the acidification of the biofilm and/or extraction of the biofilm fluids. Presumably, the measurements in these studies did not result in significant cell lysis. Indeed, the focus of the research and corresponding hypotheses strictly adhered to extracellular ion concentrations within the biofilm; that is, the uptake of ions by biofilm constituents was not considered, with a few exceptions [77]. Our previous study showed that dental plaque contains substantial numbers of polyP-accumulating bacteria. The uptake of P_i_ to synthesize polyP may be one mechanism that contributes to the P_i_ drawdown observed in biofilm fluids.

In our experiments, the addition of the fermentation substrate glucose, as well as the metabolic product/substrate lactate, both stimulated polyP accumulation by *L. rhamnosus*. Although untested at this time, it is conceivable that the accumulation of P_i_ and polyP by oral bacteria supports the PEP-PTS systems in response to sugar starvation. Our ^31^P NMR data revealed an unexpected finding that may not have been previously considered to play a role in the virulence of oral LAB strains. In model LAB, intracellular P_i_ is a regulator of glycolysis and is present in low concentrations during active glycolysis. However, P_i_ accumulates during hexose sugar starvation, poising the cells for sugar uptake via the PEP-PTS system. However, the genera of the model strains in which PEP-PTS has been studied, *Lactococcus lactis* and *Streptococcus pyogenes*, lack the gene repertoire for polyP accumulation [14]. Interestingly, some evidence suggests that the members of these genera, including the oral isolate *Streptococcus sobrinus* SL-1, may accumulate polyP [82,84] by an unknown mechanism [85] in the form of short-chain polyP [86]. In *Lacticaseibacillus,* the accumulation of P_i_ in the form of long-chain polyP during glycolysis may provide a mechanism for intracellular P_i_ accumulation that would not inhibit glycolysis. Moreover, the accumulation of intracellular P_i_ prior to starvation may leave polyP-accumulating LAB better poised to respond to intermediate influxes of sugar and to take up greater amounts of P_i_ than non-polyP-accumulating LAB strains.

More generally, the accumulation of P_i_ by both non-polyP- and polyP-accumulating LAB may play an unrecognized role in oral virulence by removing phosphate, potentially generating undersaturated conditions in oral fluids during the consumption of sugar, which is the most important promoter of dental decay. However, in one oral biofilm study, substantial P_i_ was depleted from the biofilm matrix within 5 min after a sugar challenge [77]. Interestingly, these experiments promoted the growth of Lactobacillaceae [87]. In such cases, the uptake of P_i_ would not be correlated with sugar starvation.

We also hypothesize that P_i_ would be taken up by microorganisms to provide fuel for polyP glucokinase [88] and/or as a stress response to acidity, as reported previously [24,77]. For example, an oral strain that possesses a polyP glucokinase (ppkG NZ_LT593929.1) is *Propionibacterium freudenreichii* (formerly *P. shermanii*). This strain accumulates long-chain polyP when grown on lactate-supplemented medium. However, when grown on glucose, polyP is present at low levels as short-chain polyP [89]. *L. rhamnosus* lacks a polyP glucokinase. But the accumulation of polyP may be correlated with pH, as is observed in some other Lactobacillaceae studies. In such studies, polyP has been proposed to balance the charge of intracellular H^+^ ions [45,90]. Indeed, throughout our nutrient experiments, the highest concentration of intracellular polyP was observed when the pH dropped below 6, regardless of the culture growth phase. An additional role of polyP accumulation may be to drawdown ATP in support of ATPase, which is responsible for proton pumping from the cytoplasm, as reviewed by Wang *et al.* [91].

### Gallein and fluoride are reversible inhibitors of polyP accumulation

We observed that both gallein and fluoride inhibited P_i_ drawdown and polyP accumulation when *L. rhamnosus* was grown on BHI medium. However, the inhibition of P_i_ drawdown was partially to fully recoverable upon the addition of high amounts of glucose or lactate. Moreover, we observed an ~50% increase in extracted polyP and a different pattern in the growth cycle for polyP accumulation. In the case of gallein, roughly half of the phosphate incorporated into polyP stores appears to have been derived from unidentified organic compounds in the undefined medium. However, we cannot explain the mechanism(s) behind these observations. We hypothesize that the inhibition of PPK1 altered sugar metabolism and that PPK2 was invoked for polyP synthesis in these treatments. Gallein and fluoride interact directly with polyP kinases. Gallein is thought to bind to polyP kinases and promote direct substrate inhibition, probably by occupying the nt-binding pockets of both PPK1 and PPK2, despite PPK1 and PPK2 lacking homology [53]. However, gallein also binds to Gβγ subunits of heterotrimeric guanine nt (GTP)-binding proteins [92,94]. The exact mechanism by which gallein interacts with this subunit and potentially with the other α-subunits has not been fully elucidated, but it likely involves a WD40 repeat domain within the Gβγ subunit [95]. However, neither PPK1 nor PPK2 contain a WD40 domain. Indeed, Gβγ dimers are structurally diverse [96], and we found that they lack annotated protein domains common to PPK proteins. Given the diversity of domains that may interact with gallein, we were unable to ascertain the likelihood of gallein interacting with other enzymes or proteins.

Fluoride inhibits metalloproteins such as phosphatases, kinases and hydrolases, including enolase, a core enzyme in glycolysis [97]. We used STITCH, a web-based protein–chemical interaction networking tool [98], to explore possible interactions of PPK1 with other related proteins and metabolic products. Our STITCH-based networking analysis (Fig. S2) revealed the coordinated interaction of PPK1 with other proteins, such as F0F1-type ATP synthase subunits gamma and delta, as well as an inorganic pyrophosphatase. All three enzymes are known to be blocked by fluoride [61]. Notably, PPK1 uses ATP as a substrate for the production of long-chain polyP, whereas the latter product is formed by the activity of F0F1-type ATPase/synthase. ATPase/synthase, which regulates ΔpH of the cell membrane as well as intracellular pH, is also known to be inhibited by fluoride [99].

We were unable to find references in the literature regarding whether fluoride has been shown to directly inhibit PPK2 enzymes. However, they do have a metal co-factor, Mg^2+^, which could interact with F^−^. But in the case of class III PPK2s, as in Lactobacillaceae [100], they may use other metal co-factors Mn^2+^, Co^2+^, Ca^2+^ and Ni^2+^ [54], which might make them less susceptible to fluoride. Moreover, even within class III PPKs, there are considerable differences in their sequence and structure. Nor do we know how potentially prompting PPK2 to function in place of PPK1 would impact biochemical interactions.

Class III PPK2s preferentially phosphorylate nucleoside mono- and diphosphates from polyP, but the enzymatic activity is fully reversible [56]. In addition, fewer studies have shown that PPK2 can synthesize polyP *in vivo* when PPK1 was not functional. Moreover, Neville *et al.* observed that a class II PPK2 was less inhibited by gallein as opposed to PPK1 and class I PPK2s [55]. Indeed, Neville *et al.*’s *in silico* analyses of a class III PPK2 from *Meiothermus ruber* revealed a deeper nt-binding pocket than other types of PPKs. Thus, gallein may be less effective as an inhibitor of class III PPKs.

In the case of fluoride, we speculate that the ability of class II PPK2 enzymes to use more than one metal co-factor and potentially their unique topology may make this enzyme less susceptible to fluoride inhibition. If correct, the potential for PPK2 to be invoked in an attempt to restore polyP synthesis implies that polyP plays a central role in sugar metabolism in *L. rhamnosus.* However, fluoride is known to inhibit a variety of enzymes involved in central metabolism, including enolase. Enolase plays a central role in glycolysis and sugar uptake, as well as ATPase/synthase, which regulates intracellular pH and membrane ΔpH [99]. Fluoride can cause both reversible and irreversible inhibition of some of these enzymes. Thus, the influence of fluoride exposure on metabolism broadly, of which polyP synthesis/hydrolysis is a part, remains complex and incompletely understood. Future investigations of the role of fluoride and gallein in other strains and species beyond the single strain studied here will be needed to better understand the influence of these putative inhibitors on polyP synthesis in oral taxa.

### A dual role of fluoride for mineral solubility

The solubility of a mineral, such as dental enamel, refers to its propensity to dissolve or precipitate when in contact with a solution. The solubility of dental enamel is thermodynamically governed by the saturation state of saliva or dental plaque fluid with respect to the ionic constituents that comprise the enamel. The saturation state is dependent on the product of the activities (i.e. functional concentrations) of the constituent ions in solution relative to the K_sp_ (solubility product) of the solid phase (e.g. the inorganic portion of the enamel). In the oral environment, the mineral portion of the tooth can refer to different mineral phases including imperfect calcium phosphate minerals resulting from ion substitutions in the mineral lattice. However, for purposes of modelling solubility, dental enamel is commonly approximated as the calcium phosphate minerals calcium hydroxyapatite and fluorapatite [101]. At saturated conditions (Ω=1), neither mineral dissolution nor mineral precipitation is favoured. Under supersaturated conditions (Ω<1), mineral precipitation is favoured, whereas undersaturated conditions (Ω<1) favour the dissolution of the mineral. Saliva is commonly supersaturated with respect to hydroxyapatite; however, some individuals experience extensive mineral dissolution, while others accumulate dental calculus (mineralized plaque). Dental plaque fluids that are in equilibrium with the tooth surface can be undersaturated with respect to the mineral, either through the production of acid via the metabolism of sugars by bacteria [102] or by the uptake of phosphate by bacteria [14]. These mechanisms may be synergistic, as acid-induced dissolution releases phosphate from the tooth surface, resulting in saturated conditions that do not favour additional dissolution. However, if phosphate is released from enamel dissolution and taken up by bacteria, the cessation of dissolution may be inhibited.

Fluoride supplementation for combatting caries formation is based on the principle that in a solution that oscillates around equilibrium, the reprecipitation of enamel and substitution of hydroxyl ions with fluoride ions will lead to heterogenous formation of fluorapatite at the enamel surface. Fluorapatite is less soluble than hydroxyapatite and is thus less susceptible to caries formation. Experimental models have shown that caries are prevented at fluoride concentrations of <2.6 µM [101].

We modelled the mineral saturation state of plaque fluid under conditions with 2.6 µM fluoride vs. those without fluoride. We also illustrated the influence of the phosphate derived from our *L. rhamnosus* P-uptake experiments. Under conditions when calcium concentrations are relatively low, and fluoride is present, our modelling results show that fluids remain above saturation at phosphate concentrations of ~8.5 mM. Under conditions where fluoride is not present, the fluid is undersaturated with respect to fluorapatite at these calcium and pH conditions. The uptake of orthophosphate by polyP-accumulating bacteria can shift the solubility conditions from supersaturated (favouring mineral precipitation/remineralization) to undersaturated (favouring mineral dissolution/demineralization). The presence of fluoride not only alters the saturation state of the solution towards one of stability for the mineral phase (orange vs green lines), but our results also show that fluoride decreases the amount of phosphate taken up by polyP-accumulating bacteria. This illustrates a potential second mechanism by which fluoride may be protective to tooth enamel. However, the potential rescue of fluoride inhibition by glucose and lactate observed in our investigation further complicates the potential influence of fluoride on polyP accumulation in the oral environment.

It should also be noted that in the actual oral environment, tooth dissolution is caused by multiple episodes of exposure to conditions that favour tooth dissolution, and an important component of carries formation is dissolution rate, which is distinct from thermodynamic predisposition to dissolution [101]. Nevertheless, a typical oral fluid saturation state can oscillate above and below saturated/equilibrium conditions, and the sequestration of phosphate by polyP-accumulating oral bacteria can contribute, just as acid production does, to conditions that favour dissolution. Conditions that promote polyP accumulation may promote dissolution, whereas conditions that inhibit polyP accumulation, such as certain types of fluoride exposure, may reduce or ameliorate enamel dissolution.

## Conclusion

Oral Lactobacillaceae are commonly cariogenic and have the potential to accumulate polyP. Our findings are consistent with those of decades of oral research showing that sugar metabolism promotes dental decay. However, we propose that bacterial polyP accumulation and the concomitant reduction in the saturation state of oral fluids can co-occur and be synergistic with acid production, which is influenced by metabolic substrate availability. We found that both fluoride and gallein suppressed the uptake of P_i_ from the media and concomitant intracellular polyP accumulation, to some extent. Our modelling results show that fluoride acting to reduce the P_i_ uptake by polyP-accumulating bacteria may provide additional protection against enamel dissolution beyond the well-known influence of fluoride on fluorapatite substitution decreasing dental enamel solubility. However, exposure to high concentrations of fermentable substrates promotes polyP accumulation and restores the inhibition of P_i_ uptake by these distinctly different types of inhibitors, thus illustrating the potential complexity of polyP metabolism in the oral environment.

## supplementary material

10.1099/mic.0.001519 Uncited Fig. S1.

## Abbreviations

BHI: brain heart infusion
IMG: Integrated Microbial Genomes
LAB: lactic acid bacteria
LB: Luria–Bertani
PEP: phosphoenolpyruvate
2-PGA: 2-phosphoglycerate
3-PGA: 3-phosphoglycerate
P_i_: inorganic phosphate
polyP: polyphosphate
PTS: phosphotransferase system
