# Supplementary material for: Fluoride and gallein regulate polyphosphate accumulation in dental caries-associated Lacticaseibacillus
Source: Microbiology (Reading). 2024 Nov 28;170(11):001519. doi: 10.1099/mic.0.001519 (PMC11604172; doi:10.1099/mic.0.001519)
Supplement: Uncited Fig. S1. [file mic-170-01519-s001.pdf]

Supplemental information: Nutrient stimuli and potential inhibitors modulate polyphosphate accumulation in dental-caries-associated Lacticaseibacillus

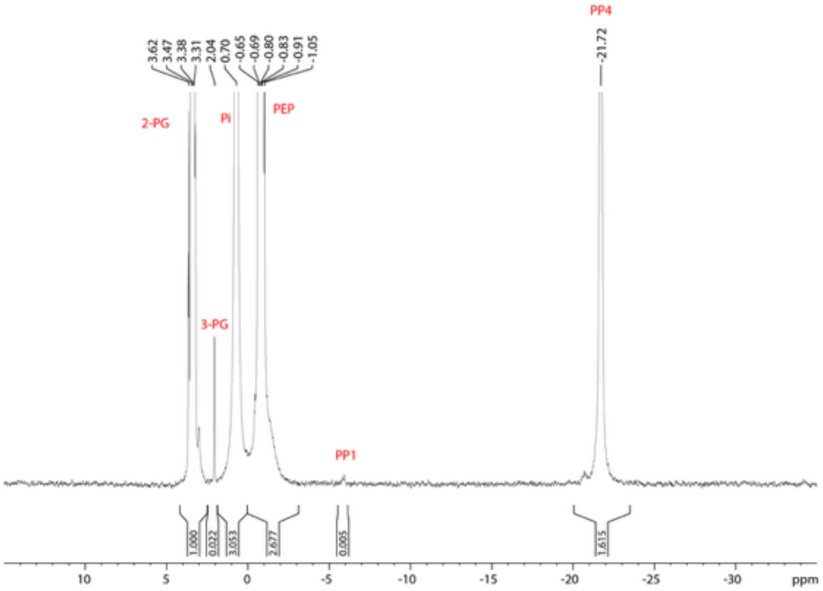

Fig S1. *^{31}P NMR spectra for polyP extracted from L. rhamnosus during late exponential growth phase. The growth medium was a modified MRS medium containing glucose.*

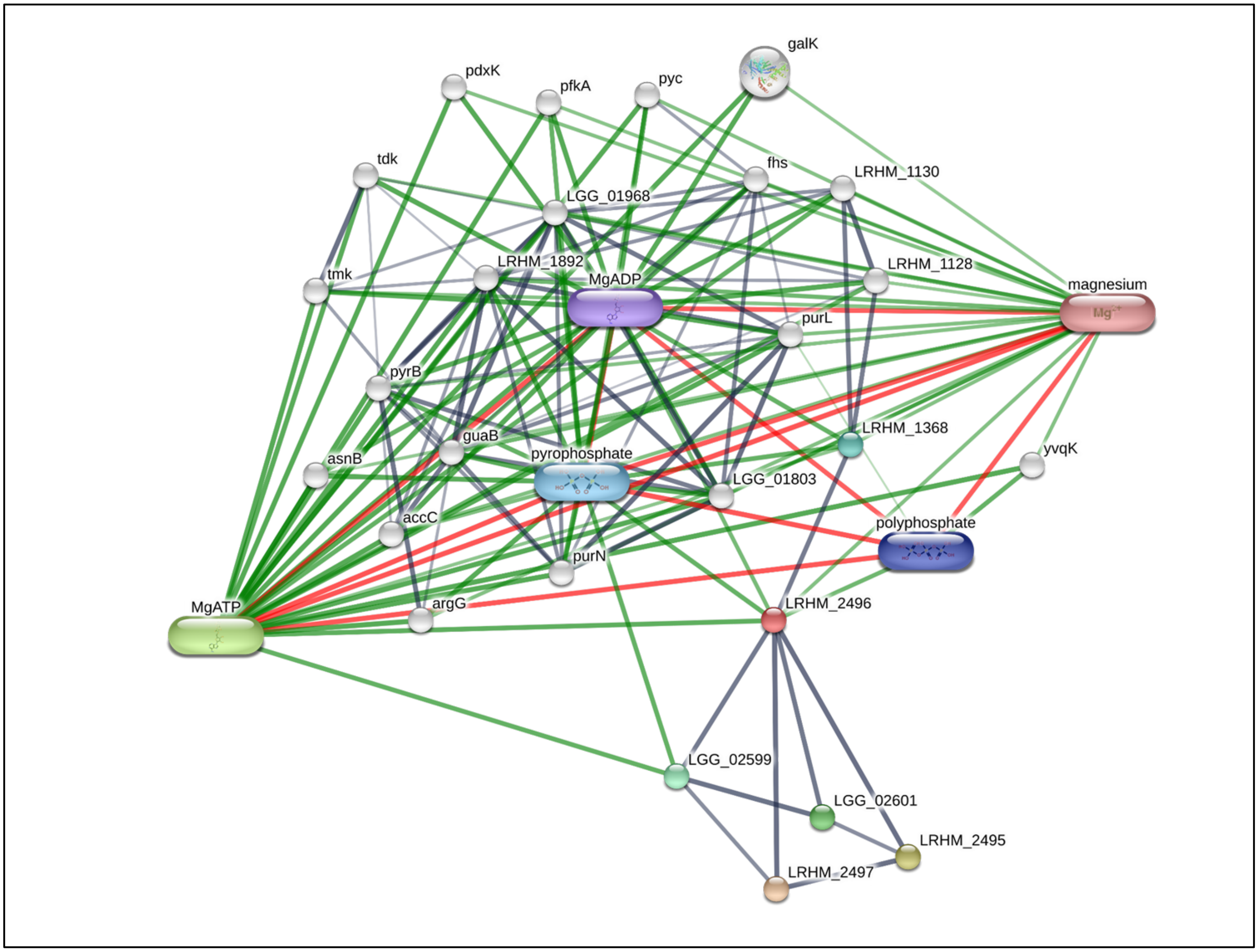

Fig S2: STITCH derived protein-chemical interaction networking image showing possible interaction of PPK with other proteins & chemicals. **galK**=galactokinase; **pfkA**=6-phosphofructokinase; **LGG_02601**= exopolyphosphatase; **LGG_02599** = exopolyphosphatase; **LRHM_1892**= GMP synthase/glutamine amidotransferase; **accC**= acetyl-CoA carboxylase biotin carboxylase; **LGG_01968**= GMP synthase; **guaB**= inosine-5'-monophosphate dehydrogenase; **LGG_01803**= phosphoribosylamine--glycine ligase; **pdxK**= pyridoxal/pyridoxine/pyridoxamine kinase; **pyrB**= aspartate carbamoyltransferase catalytic subunit; **Pyc**= pyruvate carboxylase; **LRHM_1130**= F0F1-type ATP synthase subunit gamma; **LRHM_1128**= F0F1-type ATP synthase subunit delta; **LRHM_1368**= inorganic pyrophosphatase; **LRHM_2496**= PPK.

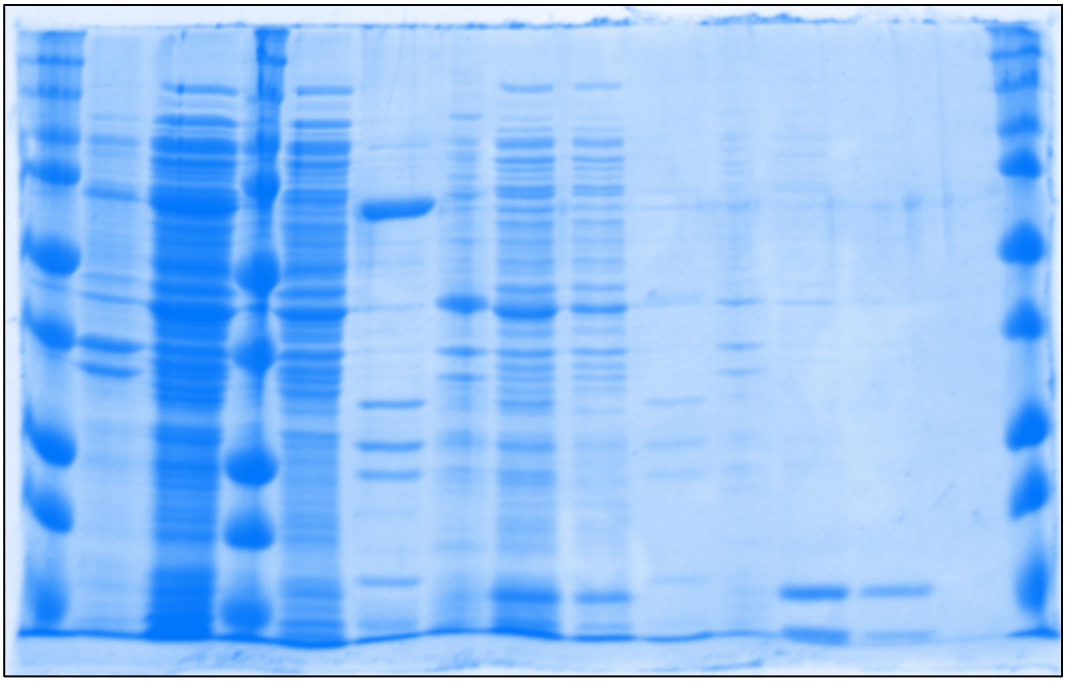

Fig. S3 – SDS PAGE gel of ScPPX.

Lane 1, 6, 15 - Precision+ Protein™ Dual Color Stds (BioRad)
2 - ScPPX Pellet in 2% SDS
3 - ScPPX lysis solution
4 - ScPPX Flow-Through
5 - ScPPX Elution
7 - ScPPX2 Pellet in 2% SDS
8 - ScPPX2 lysis solution
9 - ScPPX2 Flow-Through
10 - ScPPX2 Elution
11 - EcPPK Pellet in 2% SDS
12 - EcPPK lysis solution
13 - EcPPK Flow-Through
14 - EcPPK Elution
